# Medical Transactions of the College of Physicians, Vol. VI

**Published:** 1820-12-01

**Authors:** 


*Medical Transactions published by the College of Physicians in London.* Volume the Sixth. Octavo, pp. 426, three plates. London, 1820.

The desire of *communicating* knowledge appears to be as strongly and instinctively implanted in the human mind, from the earliest infancy, as that of *acquiring* the same. We see the child fly to its parents or companions to impart the newly-acquired idea or information; and in every stage of existence, from the cradle to the grave, we find that nothing but avarice, fear, or some base passion, can prevent man from freely communicating to others, what may have cost himself years of laborious study or research to ascertain. This is certainly no unfavourable trait in the human character; and in no class or profession is it more conspicuous than in the practitioners of medicine, where, at the same time, the most powerful temptations and inducements exist to deface it. The formation of societies, and the publication of transactions, through the medium of which, each individual may contribute that which has most attracted his attention or occupied his thoughts, are of incalculable service to the general progress of the healing art. They prevent the unnecessary multiplication of books, by giving an easy vent to facts, observations, or essays, which might otherwise expand into separate volumes, without any increase of solid materials; while, at the same time, they rescue from oblivion, or the narrow circle of individual possession, many valuable facts and suggestions which, but for these accessible vehicles of record, would inevitably perish. Volumes of this kind must necessarily be very unequal in themselves, since chance and a thousand fortuitous circumstances operate on the nature, as well as on the current of the communications that compose them. Nothing then, in our opinion, can be more unfair than to estimate the general character of a work of this description by the insulated articles of a single volume—and still less justifiable is it to measure a contributor's genius, talent, or information by the importance or magnitude of a detached paper that is but little calculated to afford any criterion, pro or con, of a man's ability, judgment, or experience. Such, however, is by no means an unusual practice in this athenian age of colloquial criticism, where we exercise the noble liberty of speech and press with unbounded latitude.

A volume issuing from the Royal College of Physicians, the highest constituted body in the profession, endowed with chartered powers, high privileges, and distinguished rank, offers a prominent object for the eulogies of friends, and the aspersions of enemies, besides affording a prolific topic of criticism for the gossips and small wits of the profession. It is not, however, for the zealous and unbiassed cultivator of medical science, or the humble inquirer after truth, wherever that may be found, to mingle party spirit with his pursuits, or suffer the distorting medium of prejudice to come between him and the objects of his research.* On this account, we shall adhere, with more than usual rigour, to the analytical line of our policy, conscious that, under existing circumstances, our criticisms would, by one party, be con-

* In truth, we conceive that the character of the college, as a body, is no more concerned in the character of a volume of the transactions published under its auspices, than our own character is involved in that of the works which we happen to analyse for our journal. If a council of the college superintend the publication, and if that council admit bad papers and reject good ones, then the judgment or impartiality of the council may be impugned. In no other way, we imagine, can the college be concerned in the work.

sidered *presumption*—our commendations, by the other, *adulation*. In laying an impartial portrait of each article before the reader, however, we shall occasionally hazard a practical remark or observation, which we trust will be received in the same spirit of candour with which it is delivered. "Interea vero, quicquid falsi vel incerti eidem insit, si artem medicam promovere cupimus, firmoque et perenni fundamento stabilire, refellere et corrigere decet, probe memores, sapientiam esse primam, stultitia caruisse."*

The volume before us contains sixteen articles, and we shall take them in the order in which they stand.

---

### I.

*History of a Case of Strangulated Hernia, successfully treated by the application of ice, after the attempt at reduction by the taxis had failed.* By G. D. YEATS, M. D. F. R. S. Fellow of the Royal College of Physicians, &c.

THE great danger to be apprehended from the incarceration of a portion of intestine or other internal part, is inflammation and its consequences. The *obstruction* to the fæcal passage is quite a subordinate consideration, excepting as it may increase the inflammation. Cold has a great effect in restraining this inflammatory tendency, not only by constringing the vessels, but depressing the nervous excitement of the parts. It is therefore very generally employed in aid of the taxis by our best surgeons. The case related by Dr. Yeats was a young man, ætat. 18, who was seized with sickness, vomiting, and great pain in the right groin and back, on the morning of the 16th March, 1807. Dr. Y. saw him in two hours after, and found a considerable swelling in the groin, protruding into the scrotum, "produced by a strangulated hernia of the congenital kind." It was extremely painful to the touch, and very tense, with great soreness over the abdomen. The countenance was pale and sunk, the pulse 100, full and hard, with thirst and restlessness. Twelve ounces of blood were taken from the arm; twelve leeches were applied to the tumour, and the bites encouraged to bleed by fomentations. By these means he obtained great relief. Five grains of calomel and one of opium were exhibited, and one grain of the former directed to be given every hour afterwards. When the flow of blood by the leeches had ceased,

* Gregory's Conspectus.

pounded ice was applied to the tumour, and in the afternoon a clyster was administered, which brought away somefæces. "The pain was by this time mitigated, and the inflammation and tumour were certainly less, so that he could lie horizontally with comfort." Mr. Macklin, state surgeon to the Lord Lieutenant of Ireland, was now called in, and attempted the reduction of the hernia, but without success. A grain of opium was directed at bed-time, and the calomel and ice to be continued as before. On the following morning the pulse was calmer, and the countenance improved. The tumour also was diminished in bulk, and the abdominal tension almost entirely gone. The same remedies were continued, and a purgative clyster ordered. "In the evening the clyster had produced a feculent evacuation, and by bed-time the hernia had returned into the abdomen with the testicle also." The testicle descended the following day; and as soon as the hernia was decarcerated, two or three spontaneous feculent evacuations took place.

Dr. Yeats makes several practical remarks on the utility, abuse, and action of cold, in strangulated hernia, which deserve the surgeon's attention. He properly disapproves of purgatives given by the mouth, before the obstruction is beginning to yield, and even then they are seldom necessary. "The administration, however, of laxative clysters is obviously required." They should not, Dr. Yeats thinks, be made with acrid ingredients. He directs the submuriate of mercury in small doses of a grain, frequently repeated, "with a view to its relaxant effect, which it powerfully exerts when it affects the constitution." We shall conclude with the following extract from this intelligent physician:

"I may here mention, although I cannot now urge the reasons in detail, that I have almost uniformly found the combined exhibition of submuriate of mercury and opium, one of the best means of arresting the progress of inflammation, especially under circumstances when that morbid condition continues after repeated general bleeding, and when it seems to bid defiance to all you can do, by lessening the general momentum of the blood. In ileus, acute rheumatism, pleurisy, and all purely inflammatory diseases, this combination, not omitting, of course, depletion, has pretty faithfully served me." P. 15.

### II.

*Some Observations upon Paraplegia in Adults.* By MATTHEW BAILLIE, M.D. F.R.S. L. and E. Fellow of the Royal College of Physicians, &c. &c.

THERE is not a more difficult point in pathology and practice, than that of ascertaining the actual seat or cause of disease. An irritation in one part will cause pain or some morbid phenomenon in a distant part, and these deceptious manifestations are not obedient to any hitherto ascertained law of the animal economy. An irritating body or secretion in the digestive organs will, in ten different individuals, exhibit ten different effects, in kind as well as situation. We have, at present, no law by which we can accurately trace the chain of causation; and therefore our great business is, to collect facts and observations, as a basis for future medical legislation.

The experience and knowledge of a man, in our profession, are generally in an inverse ratio to the boldness of his conclusions, the absoluteness of his dogmata, and the positiveness of his assumptions. The personal conduct and public writings of Dr. Baillie afford an excellent commentary and illustration of this text. Cautious in his practice, guarded in his prognosis, candid in his opinions, and, what is more estimable than all—charitable towards the failings and discrepancies of his brethren, this veteran physician offers an example for the humble to admire, and the proud to imitate. We know that in stating these sentiments, we but re-echo the general voice of the profession; yet we deem it our duty to do so, (not from any private feeling, for we owe Dr. Baillie nothing but the homage of our public esteem) because we believe that moral worth, in the higher ranks of the profession, conduces not less to the vital interests of that profession than splendid attainments. The cry for reform has been long and loud in medical society; but we apprehend that the great subject for reformation is our own conduct towards each other. The work must begin at home. When we determine to do strictly unto our brethren as we would be done to by them, harmony and unanimity will prevail, and the science will rise in public estimation. This is the line of conduct which a Baillie *has* pursued; and it is that which every member of the profession *may* pursue.

Our experienced author conceives that paraplegia has "increased considerably in this country within the last fifteen or twenty years," for which he is unable to assign a satisfactory reason. The paraplegia of adults, Dr. Baillie observes, has been considered by most medical men as being produced by some disease, "either in the bones or ligaments of the spine, or in the cavity of the spine, most commonly at the loins, independently of any disease in the brain."

"In adults, however, where there has been no accident affecting the spine by outward violence, paraplegia, I believe, depends most commonly, in a great measure, upon a disease affecting the brain itself." P. 17.

Dr. Baillie is joined in this opinion by Sir Henry Halford, Sir James Earle, Mr. Copeland, and some others of his friends. We conceive that our continental brethren have taken a pretty similar view of the pathology of this distressing disease. They consider it to result from something which interrupts the nervous energy from flowing along the spinal cord to the nerves of the lower extremities.

"Il en resulte que les mêmes causes qui produisent la paralysie en general, sont susceptibles de donner lieu à la hemiplégie elle-même."*

We need not state that the general causes of hemiplegia are pressure on the brain. In the paraplegia of adults Dr. Baillie observes that we can rarely detect any external mark of disease in the lumbar region of the spine, although thickening of the theca vertebralis, serous effusions, or other organic changes, may, and no doubt do, occasionally, take place. Still he thinks that these causes less frequently produce the disease than has been imagined, and that pressure on the brain is the more usual agent.

"In such cases the diseased condition of the brain may immediately affect a portion of the spinal marrow, and its nerves, in the same manner as it affects the nerves of a lower limb in hemiplegia; or if there be effusion of serum between the membranes of the brain, which is a very common occurrence, a portion of the serum may fall into the theca vertebralis, and press upon the lower part of the spinal marrow."† 19.

Paraplegia occurs most commonly at and after the middle period of life, and oftener in men than in females. Some affection of the head usually precedes and accompanies the disease, as pain, giddiness, drowsiness, impaired vision, defective memory. Occasionally there is numbness, or other indication of approaching paralysis in the upper extremities,

---

* Dict. de Sciences Med. Vol. 39, p. 277.

† The French include in the etiology of paraplegia, irritation of the spinal marrow, whether idiopathic or resulting from gouty or rheumatic metastasis; also its sympathetic affections produced by derangement in the stomach and bowels—a very common cause say they.

without any appearance of disease in the cervical part of the spine. The paraplegia creeps on with, at first, a stiffness or awkwardness in the motions of the limbs, increasing to a want of power to preserve the due balance of the body without the assistance of a stick. As the disease advances the stream of urine becomes more and more feeble, and at length passes away involuntarily. The bowels are generally costive, and at length, from a want of power in the sphincter muscles of the anus, the fæces are evacuated unrestrained by the will. Patients in this state sometimes live for years; but more commonly the symptoms increase gradually, and the constitution becomes exhausted. In a few instances recovery takes place.

In one of the most strongly marked cases which Dr. B. had ever seen, the bones of the scull, more especially at the sutures, were more vascular than usual; the dura mater was natural; the vessels of the pia mater were very much loaded with blood, and there were effusions of serum between the different membranes of the brain on both sides of it. The tunica arachnoides was opaque and much thickened; the substance of the cerebrum was considerably softer than natural. About four ounces of water were found in the lateral ventricles of the brain, and a considerable quantity of water was discharged from within the theca of the spinal marrow.

Dr. Baillie thinks it not improbable that, in those cases of paraplegia in which the brain is diseased, the morbid affection extends to both hemispheres.

In respect to treatment, our author found those means most successful which were directed to relieve the head, viz. cupping the nape of the neck, leeches to the temples, blisters to the nape of the neck, setons, &c.

"The internal remedies which I have used with most advantage are calomel, or the pilula hydrargyri, combined with squills, together with purgative medicines. A grain of calomel, or five grains of the pilula hydrargyri with one grain of dried squills, have been directed to be taken every night for many weeks. The purgative medicines have consisted chiefly of neutral salts, and sometimes of pills composed of the extractum colocynthidis compositum, with an addition of jalap." 25.

In a few instances advantage was derived from friction of the lower extremities with the hand, for an hour twice a day. In one case electric sparks drawn from the lower limbs produced much benefit. In those cases where the paraplegia evidently depends on disease of the bones or ligaments of the spine, the foregoing treatment, of course, does not apply. Where lead is the cause, the Bath waters have been found peculiarly serviceable.

M. Chamberet, who has written an article on paraplegia, in the *Dictionaire de Sciences Medicales*, very properly observes that the farrago of heating and stimulating remedies used formerly in paralysis, must have done much mischief. He recommends cupping and leeching the spine, while counter-irritation is kept up at a distance, to draw the irritation from the brain and spinal marrow. In the mean time, as many cases are purely symptomatic of gastric and other derangements of the abdominal viscera, he judiciously directs the attention of the physician to the remote source of the malady, rather than to its apparent seat. These observations coincide with, and corroborate the opinions of our own illustrious pathologist, as delineated in this analysis of his paper.

---

### III.

*Observations Medicales.* Par Jos. Rom. Louis Kerckhoffs, M.D. &c. &c.

The first of these observations, is a memoir and some cases of plica polonica, which, however, we shall not stop to analyse, as it is a subject but little interesting to the British practitioner, and our principle is invariably to select what is *most* useful. We may remark, *en passant*, that our author comes to the conclusion that the disease in question is entirely owing to want of cleanliness, together with the habit of wearing the hair long and constantly covered, so common among the lower classes of the Polonese. This opinion is contrary to that of the best Polish physicians. We shall therefore leave the doctors to settle the dispute. The second section is on the subject of willow bark in "phthisie muqueuse." This we have touched on in our electic article in the present number of the journal. The third section is written in Latin, and contains the case of a soldier poisoned by the acetate of lead. The quantity taken, and the time of taking the poison, are not stated. The symptoms were constipation, anorexy, great lassitude and debility, distention of the abdomen, followed by retraction of the same, sense of suffocation, nausea, convulsions, cold sweats, trismus. The stomach was found inflamed, and some portions of the mucous membrane macerated, especially about the pylorus. Spots of inflammation were also observed in the œsophagus, duodenum, transverse arch of the colon, pancreas, mesentery, liver, &c. About six ounces of a dark-coloured liquor were found in the stomach, which, on chemical examination, was found to contain acetate of lead.

In the 4th section is an opinion respecting the origin and prevention of hospital gangrene. Our author is convinced that it owes its existence to the same morbific miasm which occasions putrid fevers, and that it is to be prevented by scrupulous attention to cleanliness in the beds, &c. of the wounded, and free ventilation.

The 5th section or observation is on scabies, which Dr. K. has long been in the habit of curing by a solution of muriate of soda two ounces, in a quart of infusion of arnica montana, which is prepared by pouring two pints of boiling water on four ounces of the flowers or roots of arnica. With this liquid the affected parts are to be washed three or four times a day. In the 6th section Dr. K. strongly recommends fomentations of hot water containing carbonate of potash, in cases of whitlow, especially when suppuration has evidently commenced, and the proper incision has been made. We do not consider the addition of the alkali as of any great importance.

The latinity of these articles has been objected to by some of our classical friends. To us the language has clearly conveyed the author's meaning, and beyond this we do not look, in the simple narration of facts or observations.

---

### IV.

*Description of an unusual Appearance in the Viscera of an Infant, &c.* By Henry James Cholmeley, M. D. Fellow of the Royal College of Physicians, &c.

The infant was observed to be very sallow on the day after birth, which appearance daily increased till it became completely icteric. The meconium was scanty, but of the usual appearance. An emetic brought away some dark green bile, and the yellowness of the eyes and skin diminished. The stools continued white and pasty, the urine of a deep hue. At the end of five weeks, during which the jaundice continued, an attack of convulsions terminated the child's existence. The infant took the breast freely while it lived; but vomited up what it took. The bowels were seldom moved without medicine. On dissection, the liver was found of a natural size, but of a green hue throughout its substance. The gall bladder was totally wanting, there being only a narrow impervious cord in its place, with a small knot at each extremity. The pancreas was enlarged and indurated, so as to obstruct the egress of bile from the liver to the duodenum by the ductus communis choledochus. This circumstance prevents our gathering any thing from the case relative to the use of the cystic bile; since all the phenomena recorded might readily be attributable to the interrupted course of the bile generally into the intestines. The case clearly shews, however, the importance of the biliary secretion, and the effects of its obstruction.

---

### V.

*Remarks upon the Effects of a Warm Climate in Pulmonary Consumption, and some other diseases.* By H. W. Carter, M. B.

Dr. Carter's observations on this subject, are very congenial with those lately promulgated by Dr. Clark of Rome, whose work we reviewed in the 8th number of our quarterly series.

Dr. Carter thinks, and with reason, that persons labouring under pulmonary affections are too indiscriminately advised to a warm climate. They are often sent out when their complaints are hopeless, to pine and die in a foreign land! On the other hand, "the very first appearance of affection of the lungs, should be attended to; and, provided there be any predisposition to consumption in the patient, and his circumstances be such as to render a journey to the South practicable, a mild climate should be recommended without delay." Our author properly observes that it is much better that the patient should be frightened, than lulled into a false security. He has seen decided advantages from a warm climate in incipient phthisis. But invalids, he remarks, are not to imagine that a single winter's residence abroad will be sufficient to restore to health a person whose lungs are affected. It will often be found necessary to continue several years out of England—in some instances, the whole of the person's life. He has known some fatal consequences from returning prematurely to England. From all Dr. Carter has been able to learn, "it is only in cases of incipient phthisis that a warm climate is of any advantage."

"When the disease has assumed a marked character, when there is much cough, with purulent expectoration, flushing, and a frequent pulse, and emaciation, I believe that climate never effects a cure, and very seldom prolongs the existence of the sufferer." P. 64.

In such cases, indeed, we ourselves and many others have seen the disease hurried on, as the warm latitudes were approached. M. Risso, a gentleman of Nice, is now employed on a work to prove that residence on the *sea-coast* is injurious to consumptive patients. We suspect that the disadvantages are owing to something in the topography of the place; rather than to its vicinity to the sea. No example of recovery from confirmed phthisis has occurred at Nice, by our author's account—nor in many other places, we fear! Yet he says the climate of Nice is as good as that of Lisbon or Naples, while it is superior to that of Marseilles or Montpellier, where patients suffer much from the north and north-east winds. And, as it is pretty evident that climate has no power in checking phthisis, after it has passed the first stage, it is to be remembered that the distress, occasioned by travelling under such circumstances, must generally aggravate the symptoms. "More mischief is done by the journey than the finest climate can repair." Besides, if the patient do struggle through the winter in Italy or the South of France, he is obliged to move again in the summer, to avoid the intemperate heat; and thus travelling soon becomes irksome to an invalid, especially where the accommodations are so bad as in the countries in question.

In the *early stage* of phthisis Dr. Carter thinks there can be no doubt of the service derived from a sea voyage, the sickness thereby induced being probably more efficacious than that from medicine. In confirmed phthisis, however, whether the sea air be injurious or not, the sickness exhausts the patient without alleviating his complaint, while the tædium and discomfort of a ship render a voyage almost as bad as a journey by land. In short, our author thinks, and so think we, that "persons in confirmed phthisis should never quit their own country."

A warm climate being allowed to be useful in incipient phthisis, the next point to ascertain is—the most eligible residence. Madeira, our author believes to be the finest climate in the world. We much doubt this. But there are many objections, particularly the mountainous nature of the country, rendering it almost impossible for the invalid to take proper exercise. In Spain, Barcelona, and Malaga, Dr. C. thinks are good winter residences, if the patient can make up his mind to see few of his countrymen, and meet with little to amuse or interest him. At Lisbon the weather is often so cold and rainy, in winter, that it cannot be recommended. Marseilles Dr. C. considers an improper place. The sun is powerful, while the north wind, when it blows, is piercing cold. What is worse than all, the changes of temperature are sudden and great. The same observations apply to Montpellier. The sea wind is said to be so loaded with moisture, that when it prevails "the furniture and beds become excessively damp." Hyeres, Dr. C. thinks, is probably the best residence in France, as to climate; but there are many objections to it. It is poor—affords but very indifferent accommodations—no society—nor medical assistance that can be relied on. Florence "is decidedly a bad winter residence." "The climate of Rome is not to be compared with that of Naples, which is, indeed, delightful." The only objection is the Scirocco, or south-east wind, which in summer is very oppressive, but in winter bearable.

"The scenery around Naples never ceases to be interesting: and the general fineness of the weather, and facility of conveyance from one place to another, enable an invalid to procure much instruction and amusement without risk or fatigue." P. 78.*

Upon the whole, our author thinks Naples an elegible winter residence; yet if the fascination of scenery and the interest of the environs of Naples be put out of the question, our author would give the preference to Nice above every other part of Italy with which he is acquainted. The walks and rides about the town are as varied and pleasant as can be desired, and the botanist and geologist may gratify their curiosity.

But wherever the invalid may fix his residence, there are certain precautions, which he should always bear in mind. He must not trust too much to the mildness of the weather, and throw off too hastily those clothes to which he has been accustomed. He ought not to be out late in the evenings. The invalid should not expose himself to the sun, and then stand in the shade; between which, in all warm climates, a great thermometrical difference prevails. He should not expose himself to the prevailing winds. In his diet he should be cautious; for although the wines are very pleasant, and not so heady as our own, they are obviously improper where the lungs are weak. The high seasoned dishes, so common abroad, are to be avoided, as well as the sauces of which the French and Italians are so fond. The fruits of the South, however, are wholesome and refreshing, especially if taken before dinner.

Neither Naples, Nice, nor any of the places enumerated by our author, are considered by him as proper summer residences for the pulmonary invalid; "some of them

* It may not be improper to say that, in Dr. Miln, resident physician at Naples, (a gentleman to whose professional character we can speak from personal knowledge) the English invalid will find a countryman, in whom he may place the fullest professional confidence.

being extremely unhealthy, and all of them too hot at that season."

"In the early part of the summer, perhaps, the banks of the Lago Maggiore, or of the lake of Como, or some spots in Tuscany, or Grasse in France, might be selected; but in July, and the two following months, the heat is too great even at these places; and it will be adviseable to move to Geneva, or somewhere in the neighbourhood of that town. There are several spots near Geneva which are reckoned very healthy, and where the climate is good in summer. Mornex is one of these. About the commencement of October the patient may return to Italy or the South of France." 86.

If the winter residence have been Lisbon, the patient should move, as soon as the hot weather sets in—say to Cintra, for example, where the country is eminently beautiful, and the air always cooling and refreshing.

Our author makes a few brief remarks, in conclusion, on the influence of a warm climate on some other diseases besides pulmonary complaints. Over gout he has seen climate exert a powerful influence. One striking instance of which he relates. A patient inherited a predisposition to, and was early and universally affected by, the disease. He was nearly deprived of the power of walking, and his stomach had become seriously deranged. During this gentleman's journey from England he experienced a severe attack of atonic gout, which confined him for several weeks. He arrived at Nice late in the autumn, and greatly debilitated, with much derangement of stomach and bowels. At Nice he continued a considerable time, and recovered not only the power of walking, but the healthy tone of his digestive organs. Chronic rheumatism and asthma are slightly noticed as being benefited by a warm and equable climate. Dr. C. informs us that it is now fashionable to send patients abroad for deafness; but this disease has not appeared to be in the least benefited thereby.*

For further information on this subject we refer our readers to Dr. Clark's very interesting little work on climate, which should be recommended to every patient before he parts from England, as containing many valuable remarks and much important information, touching his convenience as well as health. The extent to which we have carried the analysis of this article, will shew our estimation of its utility, as well as our respect for its worthy author.

---

* We suppose it will soon be necessary to send patients to Italy, or the South of France, for blindness, corns, warts, &c.

### VI.

*On the Medicinal Properties of the Solanum Tuberosum, or Potatoe Plant.* By John Latham, M. D. F. R. S. the President.

We have many medicines, but few remedies. Of the former, many are expensive exotics, as opium for example; and therefore it would be exceedingly desirable, both in point of economy, and to check the inducements towards adulteration, if substitutes, of easy access and cheap materials, could be procured. This is leaving out of the question the chance or hope of discovering useful properties in the new articles investigated.

The potatoe, being a species of solanum, might be conceived, as Dr. Latham observes, to possess qualities analogous to others of that class; "but experiments to ascertain the fact had hitherto been wanting to fix its character as an article of materia medica." Accident led Dr. Latham to ascertain the narcotic powers of this plant on the human body, and he thinks the effects produced in the following cases "will perhaps entitle it to a rank of no mean estimation in the catalogue of medicines."

From about seven pounds of the leaves and stalks, Mr. Hume, of Long Acre, prepared for Dr. L. nearly a pound of extract, in the taste of which there was nothing peculiar that might prove disgusting to the patient. The dose, at first, was half a grain three times a day, which was soon raised to two grains at a dose.

The first patient to whom Dr. Latham exhibited the medicine, and by whom he ascertained the dose, laboured under cough, with mucous expectoration. "As much relief was obtained as might have been expected from the extract of conium, under similar circumstances. No distress was felt in the system; but the patient imagined that his bowels were rather more relaxed than was usual with him."

The second was a very old case of rheumatism, with distortion of the limbs, articular nodosities, chronic cough, and at times intolerable headache. While taking the smaller doses of the extract, the patient felt something like the tremor and disturbance sometimes produced by digitalis. The solanum was therefore suspended for a few days and again renewed. The dose was now gradually encreased to a grain every six hours, and, in a few days, to *two grains* with directions, should stupor or tremor come on, to discontinue the medicine. The tremor did come on, and the dose was omitted *once*. It was then resumed with considerable advantage, the cough and other painful affections being much alleviated. Three grains of the extract with one of rhubarb were now ordered three times a day; "and after an interval of three days I found her still freer from pain, and more comfortable, as she expressed herself, than she had been for several months." A few days afterwards, however, the tremor, with much headache, recurred and continued for some time. These went off by a dose or two of rhubarb.

The third case was that of a lady who had a small calculus lodged in the ureter. She was ordered three grains of the extract three times a day, from which she experienced much relief. In four days the piece of gravel passed into the bladder, and was afterwards voided. She was soon quite well.

We shall pass over the two next cases, as not very satisfactory, to notice the sixth, which we shall quote entire.

"A young gentleman, about twenty years of age, had been afflicted with almost constant cephalalgia for two or three years. Various had been the means employed to procure relief, without any sensible advantage; bleeding, cupping, leeches, blisters, puragtives, antimonials, mercurials, &c. had successively been tried, and afforded not the least benefit. I put him also upon several plans without any permanent relief; conium with submurias hydrargyri, steel with aloetic preparations were tried. The rectified oil of turpentine was then given in the dose of an ounce and a half at once. Some remissions were certainly experienced for an hour or two in the day, and after the turpentine, for a whole day, but then the pain recurred as usual. I now directed three grains of the extract of solanum three times a day, having indeed about a fortnight before, given him the smaller doses of one grain without any effect. After having tried the solanum in the increased dose for a week, he came to me in a much improved state, not having had any pain for a longer time than half an hour in the day, and that in a greatly mitigated degree. In a few days, however, the pain returned, and I directed the dose to be gradually increased to six grains. He could not go beyond five grains, for after each dose considerable stupor was produced for about two hours, when the pain returned, although in a less degree. He was now desirous of returning into the country, where I recommended him a plan of decoct: aloes with chalybeate wine." 102.

The last case was one of supposed angina pectoris from water in the chest or perhaps organic disease of the heart itself. Under the use of the solanum tuberosum, "his pulse certainly became regular, and he thought himself better, as he had been able to call upon me with less difficulty. He felt the same tremors from its use as were observed in other cases." 103.

Dr. Latham declines detailing several other cases, as the results were very similar to those here stated. A medical friend of his has already advanced the dose to thirty grains at a time, in a case of cancer of the uterus. Dr. L. concludes thus:

"I am unwilling to say more about the solanum tuberosum, lest I should hereafter be found to have said too much; but I think it superior to hyoscyamus and conium, and therefore with confidence recommend it to my professional brethren, not only in cases where those medicines have been most commonly employed, but generally in all chronic cases where there may be excess of painful irritation or irregularity of action." 104.

We hope those of our brethren who are attached to public institutions, will give this medicinal substance a fair trial as to its narcotic powers, in consequence of Dr. Latham's recommendation.

---

VII.

*On certain painful Affections of the Intestinal Canal.* By Richard Powell, M.D. Fellow of the Royal College of Physicians, &c. &c.

Dr. Powell justly observes that a knowledge of the causes of disease is always desirable, but not seldom obscure. Every attempt then to establish the relations between cause and effect, is deserving of attention, and will receive it from the profession.

"Whenever," says Dr. Powell, "violent pain takes place in the epigastric region of the abdomen, exacerbating in paroxysms accompanied by sickness, yellowness of the eyes and skin, and urine, by clay-coloured fæces, and without any proportionate increase of action in the circulation, biliary concretions are supposed to be forcing through the ducts; and when these symptoms abate, it is inferred that their passage into the duodenum has been effected."

It is usual, as Dr. P. observes, to examine the stools upon these occasions, though the search is often ineffectual. The biliary concretions, however, are thought to escape, or to be dissolved after they are freed from the biliary ducts. It is Dr. Powell's object in the present paper, to shew that the symptoms usually ascribed to the passage of biliary calculi "are also referable to another cause." And as in the case of biliary concretions, we may naturally expect a repetition of attacks, and no very speedy termination of the patient's sufferings, so, on the contrary, Dr. Powell conceives that "the sort of attack to be here described is more isolated, and when once it has passed, its recurrence is by no means equally to be dreaded as is that of the former."

When our author has suspected the passage of biliary concretions, he has directed the usual large pan to be filled with water, and the fæces to be stirred in it, after which the water has been left to rest long enough to ascertain whether the concretions, as they often do, have risen to the surface. This water he pours off, and repeated similar affusions are made, time being allowed for subsidence between each. The residue is then to be examined. This residue, in the cases to which Dr. Powell refers, "has exhibited a large quantity of flakes mostly torn into irregular shapes, and appearing to have formed parts of an extensive adventitious membrane of no great tenacity or firmness." In the first case that came under Dr. Powell's notice, this membrane was passed in perfect tubes, "some of them full half a yard in length, and certainly sufficient in quantity to have lined the whole intestinal canal." In the others also, the aggregate quantity has been very large, and continued to come away for many days, in irregular thin flakes, of but one or two inches in extent, and not perfectly tubular. Dr. P. has definitely examined four such cases, in all of which the leading symptoms led him to suspect the passage of biliary concretions at the time. They were all adult females. In but one of these our author had been consulted for *previous* ill health.

"She had frequently suffered from occasional pain in the intestines, and derangement of her powers of digestion, with flatulence, and a sense of suffocation. She was always relieved, at the time, by mild opening medicine, and believed herself able to prevent the attacks of pain from increasing to any serious degree of violence, by repeating it according to circumstances. A similar history of liability to frequent recurrence of pain, accompanied by indigestion, was related to me in the other instances. The more violent siezures under which I saw all the patients, consisted in a sudden and excessive pain in the epigastric region, increasing in paroxysms very frequently, rather relieved by pressure of the patient herself at the time, but leaving great soreness and tenderness during the intervals. This state continued under four days; during it the stomach was very irritable, and the tongue coated and clammy. Jaundice came on at an early period, and the stools were white, brown, or somewhat greenish, and streaked in colours, until the films began to pass, when they were mixed with a full sufficiency of bile, but not at first of a healthy colour. The pulse throughout was calm, moderate, and natural, in none of the instances amounting to 90." P. 112.

There was no indication of inflammation. In one instance Dr. Powell noticed a considerable hardness and contraction of the abdominal muscles; in another case there was superadded to the above symptoms a difficulty in passing the urine, with pain in the region of the bladder, requiring the use of the catheter.

Suspecting the passage of concretions, in these cases, Dr. P. did not order venesection or emetics.

"The practice, indeed, which appeared to me most advantageous, was the steady use of a mixture of the infusum gentianæ compositum and infusum sennæ, with the addition of from ♏.X to ♏.XX of liquor potassæ, repeated so as to produce four or more stools in the twenty-four hours. Under its use the flakes first separated, and continued to do so, in great abundance; the jaundice disappeared, and the patients recovered health and strength." P. 114.

Saline purgatives had been tried at first, but under their use the canal did not appear so completely to discharge its contents as when the medicine abovementioned was exhibited.

Dr. Powell observes that the formation of adventitious membranes in the intestinal canal has not been so frequently noticed as it has in circumscribed cavities. "The appearance which comes nearest to it, both in resemblance and situation, is the membrane formed in the trachea under croup," but the symptoms are there more violent and destructive from locality of situation. The most remarkable circumstance, our author thinks, is "the production of an effect usually ascribed to inflammatory action without its previous existence." Coagulable lymph is known to be thrown out upon the mucous membrane of the intestines in inflammation, but in the cases in question, Dr. Powell thinks there was no evidence of an inflammatory process going on, as the pain was rather of a spasmodic nature, and the skin and pulse remained natural. Our author missed the opportunity of chemically examining the flakes thus evacuated; but a quantity of them put into spirit of wine "was contracted by its action, so as to look almost fibrous rather than membraneous, as at first, by which one would be led to suppose that they may consist of albumen, though this is only a supposition." 116.

We have not interrupted the thread of the analysis, in order that we might introduce a few observations of our own at the end. We have seen several cases of the kind described by Dr. Powell, and we have experienced them personally, so that our attention has been a good deal directed to the pathology and treatment of the complaint.

Every one who has seen much of dysentery, whether in hot or cold climates, must have observed those portions of adventitious membrane (if the term be allowed) frequently come away, especially towards the close of the disease in bad or fatal cases.* The symptoms during life, and the examinations after death, have pretty clearly proved these produc-

---

* See Morgagni, 31st Epistle, for example.

tions to be the result of great irritation and inflammation in the mucous membrane of the intestines: we know too that irritating substances swallowed have caused tubular exudations to be formed and thrown off by stool, precisely similar to those we are speaking of. Tartra, for instance, relates the case of a woman who had swallowed a quantity of nitric acid, and who, on the twentieth day afterwards, passed, *per anum*, a long membraneous packet, in one entire piece, rolled up and folded on itself, which, on being disentangled, exhibited a complete mould of the œsophagus and stomach, with all their sinuosities. From the moment that this was thrown off, the internal surface of the œsophagus and stomach became so sensible and irritable that death ensued in a few days.*

"The false membranes," says Villermé, "of the mucous membrane of the alimentary canal, are perfectly similar to those formed in croup. The fragments passed by vomiting or by stool, are generally soft, pulpy, and without any appearance of organization. *The re-agents which concrete albumen render them hard.* Sometimes these flakes are considerable, and when developed in water, represent entire tubes broken off irregularly at the ends, which have led to the erroneous opinion that they were portions of the mucous membrane of the intestines. I have had occasion to see great quantities of these adventitious membranes passed by stool, in the colic of Madrid. They were generally of a gelatinous consistence, and enveloped in mucus. One of these exhibited an exact mould of the intestine, being tubular, and some inches in length." *Dict. de Sciences Med. vol.* 32, *p.* 261.

In such cases there can be little doubt of the formation of these false membranes being the legitimate product of irritation and inflammation, in the same way as they are formed on the serous surfaces, as we shewed in our article on peritonitis. But we meet with the said membraneous substances under very opposite circumstances; viz. in sedentary persons, especially females, who have languid and imperfect digestion, with a torpid state of the biliary and intestinal secretions. Here gelatinous concretions would appear to take place on the internal surface of the intestines, and even the hepatic ducts themselves, from mere remora of the fluids and want of energy in the secretory glands and organs. To this obstruction of the biliary ducts, we have traced many instances of jaundice, accompanied by all the symptoms attending the passage of a biliary calculus, as described by Dr. Powell. At the decline of the disease, we have not only seen the

* A. B. Tartra, de l'empoisonnement par l'acide nitrique, p. 169.

membraneous fragments mentioned by Dr. Powell, but we have seen substances resembling worms or vermicelli, of different sizes and lengths, and which were evidently of the same nature as those broader fragments that had lined the intestines. We conceive they had lined the hepatic ducts. The cases detailed by Dr. Powell all evinced obstruction to the flow of bile into the intestines, and regurgitation of the same into the current of the circulation, accompanied, of course, by icterous suffusion and white stools.

To those, however, who may feel difficulty in conceiving that these albuminous incrustations can be owing to mere inactivity of the secretions, we would suggest another view of the subject. Where we find defective and irregular secretions of the chylopoetic viscera, we almost invariably find the secreted fluids depraved in quality—being sometimes extremely acrid, fetid, and irritating; at others, scanty and insipid. Now, whoever has been so unfortunate as to be afflicted in this way, can vouch for the train of painful and uneasy sensations which occupy the line of the alimentary canal, while these depraved secretions are passing, and will very readily believe that such sources of irritation may be quite adequate to the production of those gelatinous exudations, which, in other cases, have been clearly traced to irritation and inflammation. That the irritation of depraved bile itself has often caused spasmodic constrictions of the biliary ducts, and thereby produced jaundice, is believed by many; and it is stated in a recent publication, on affections of the liver, that—

"The symptoms attending these spasmodic constrictions of the biliary ducts are sometimes as violent as those where calculi are impacted. Indeed it appears to be the spasm, in both cases, that causes the intense pain."*

The same author observes that:—

"The passions have a most remarkable effect on the secretion, and also on the ducts of the liver. A fit of anger will so derange the state of the bile, as to tinge the skin yellow in a few hours. *A sudden and unnatural acrimony of a secretion may very readily excite the organic contractility of a tissue composing a canal, and thus occasion a stricture of its calibre for the time being.* In this way I have no doubt that jaundice is often induced by mental emotions, as anger, jealousy, grief, &c.†

In addition to the judicious therapeutical measures detailed by Dr. Powell, in the complaint under consideration, we may

---

* On Derangements of the Liver, p. 77. † Ibid. p. 76.

be permitted to state that in the paroxysms we have found the warm bath—warm fomentations—leeches—and blisters, serviceable as external remedies; and that internally, we have been in the habit of allaying the pain and irritation first, by pretty large doses of opium and hyoscyamus, and then acting on the bowels pretty briskly by compound extract of colocynth, calomel, and hyoscyamus, aided by purgative enemata to solicit the peristaltic action.

It is of more importance, however, to prevent the recurrence of these distressing attacks; and we are convinced that this can only be effected by not only regulating the state of the bowels, but by correcting the depraved secretions themselves. This is to be done by strict regimen, great attention to the functions of the skin, and by such remedies as act on the biliary secretion. We have recently removed the tendency to this complaint in a lady who, for several years, seldom passed two months without an attack similar to what Dr. Powell describes, by alterative aperients, generally aloes, soap, blue pill, and ipecacuan, together with a steady perseverance in the use of the nitro-muriatic acid bath. She now rarely takes any medicine internally; but has recourse to the bath, when the secretions become dark or light-coloured, by which means they soon become improved. She has experienced no attack for seven or eight months past.

We think the profession are much indebted to Dr. Powell for having drawn their attention to a painful complaint whose etiology was badly understood, or absolutely mistaken in many cases, and whose treatment was consequently defective.

---

### VIII.

*Narrative of Facts relative to the repeated Appearance, Propagation, and Extinction of Plague among the British Troops in Egypt, in the Years* 1801-2-3. By JOHN WEBB, Director General of the Ordinance Medical Department.

ALTHOUGH it is highly improbable that, under existing circumstances, and in the present improved state of society, the plague shall ever revisit, or, at all events, *spread* on the British soil, yet the solid proofs of its peculiar and contagious nature, (drawn from ample experience, not insane reveries of the closet,) are deserving of record, in order, at any future time, to silence the idle dreams or visionary speculations of those who may endeavour to force themselves into notice, not by the exertion of well-applied talent, but a pugnacious attack upon some well-founded principle.

The great body of this paper is taken up with official documentary evidence of the facts mentioned in the title of the article, and which we must pass over, as not necessary to be noticed in this place. They clearly establish the contagious character of the disease, and exhibit practical examples of the means of bounding its ravages, when it unfortunately finds its way into a fleet or an army.

"So far," says Mr. Webb, "as my own experience, aided by enquiries, enables me to form an opinion on this intricate subject, I am inclined to consider the plague as much an inhabitant of Egypt, as the small-pox is of England." And again—"various other particulars occur to my mind, in confirmation of what I have advanced, but presuming those I have already adduced are amply sufficient to prove the contagious nature of the plague, I shall only beg to say, in conclusion, that if I were again so circumstanced as to have the care of preserving an army, or a community from the calamities it occasions, confided to me, I should consider no plan of precaution of any avail, if not founded on a full admission that pestilential contagion is one of the most subtle poisons that exists, as well as the most destructive to human life; and that there is no safety for strangers in the countries subjected to its ravages, whether the disease be prevalent or not, but in adopting and uniformly observing the strictest precautions to avoid the sphere of its action." 168.

It is needless to say that in these sentiments we entirely agree, with an exception to the first sentence. For as we do not consider the small pox an inhabitant of England, particularly, neither do we believe that the plague is bounded by the limits of Egypt. This would be making them local or endemic diseases, whereas we know them to be specific contagions, very capable of being transported from place to place, and even from country to country.

Our analysis of the volume under review will be continued in the next number of this journal.

